# Treatment Complexities of a Young Woman Suffering Psychosis and Pituitary Adenoma

**DOI:** 10.1155/2011/246820

**Published:** 2011-11-02

**Authors:** Maxine Sigman, Kate Drury

**Affiliations:** ^1^Inpatient Psychiatry, Jewish General Hospital, 3755 Cote St. Catherine Road, No. 542A, Montreal, QC, Canada H3T 1E2; ^2^Department of Psychiatry, McGill University, Montreal, QC, Canada H3A 2T5; ^3^Department of Psychology, Concordia University, Montreal, QC, Canada H4B 1R6

## Abstract

This paper is a clinical description of the presentation, therapy, and pharmacological management of a 28-year-old woman who had nine admissions to a psychiatry ward, the last four within one year. It became clear that the treatments, which the patient had received concurrently for ten years for a pituitary adenoma and for psychotic symptoms, were counteractive. The case highlights the importance of the role of prolactin in psychosis and of an interdisciplinary team approach when patients present with complex symptoms.

## 1. Introduction

This paper is a clinical description of a 28-year-old woman who, over a decade, had 9 hospitalizations for psychosis in psychiatry, the final four in a one-year period. This rapid deterioration led us to uncover potential interactions between her medication for a prolactin-secreting pituitary tumor and those she was receiving for psychosis. The clinical effects of increased prolactin levels are poorly understood; however, there is some evidence that increases in serum prolactin may be associated with several psychiatric disorders [1], and conventional neuroleptic medications may cause an increase in serum prolactin levels [2]. Dopamine antagonists are used to treat psychotic symptoms, while dopamine agonists are used to treat prolactinomas. As a result of the potential counteractive nature of the treatments, effective management of individuals who present with both problems is extremely difficult. A lack of guidelines regarding the monitoring for hyperprolactinoma in patients receiving antipsychotics only serves to exacerbate the problem [3]. The case presentation will underscore the importance of teamwork and integrating psychiatric and somatic treatments on a psychiatric ward.

## 2. Case Presentation

### 2.1. Psychiatric Treatment

Ms. L was first hospitalized at the age of 18. She was in a catatonic state, her chin was hairy, and she was circling her fingers repetitively. The following day she was able to speak but was bizarre and inappropriate, believing she had been admitted because she was cruel to animals as a child. Ms. L reconstituted quickly, and after two weeks on the antipsychotic drug olanzapine 10 mgs at bedtime (qhs), she gave the following history. She had been feeling ill only during the past year when her mother, who suffers from schizophrenia, had a long hospitalization following a divorce from Ms. L's father. Ms. L was not a substance abuser. She had lived with her father who, she explained, had previously physically, sexually, and emotionally abused her. Ms. L had had severe hirsutism for two years. Her prolactin level was 125.1 units (normal range: 20–25 ng/mL). Her diagnosis at this point was psychotic episode, post traumatic episode had to be ruled out. Magnetic resonance imaging (MRI) of the brain was ordered and revealed a pituitary tumor. An endocrinologist prescribed 5 mg bromocriptine to shrink the microneuroadenoma. 

Ms. L's second admission was 6 months later when she expressed feelings of suicidality to the occupational therapist at her group home. An interview with a consultant revealed no Axis 1 diagnosis. She was discharged with a diagnosis of troubled grief reaction and prescribed olanzapine 20 mg qhs, paroxetine 20 mg daily (qd), procyclidineHCl 5 mg twice a day (bid), and, in addition, bromocriptine 5 mg qhs to treat the pituitary tumor. She was to go to a rehabilitation program and see a therapist in the youth service. Some time later, she left for the Middle East, where she had one admission to hospital for a psychotic state. On her return to North America, she was diagnosed by our outpatient department with schizophrenia and treated with risperidone. She continued to have hirsutism and to be treated by her endocrinologist with bromocriptine.

On Ms. L's fourth hospital admission, she was preoccupied with religious ideas and had minimal insight and judgement. She was prescribed 16 mg risperidone qhs and quetiapine 200 mg in the morning (qam) and 400 mg qhs for her psychosis. The following year Ms. L was again admitted psychotic and anxious, hyperventilating, showing poor eye contact, and moving her fingers in a circular motion. She had told her parents she had felt paranoid and suicidal. Again her insight and judgement were poor. She had not complied with the psychiatric follow-up treatment as her mother, in her own psychotic state, had cancelled it. In hospital, Ms. L remained psychotic on 100 mg of loxapine and 500 mg quetiapine. She was given electroconvulsive therapy, and this seemed to help her. She agreed to placement in a group home and to take her medications on discharge. Ms. L was prescribed flupenthixoldecanoate 75 mg q 2 weeks I.M., divalproex sodium 500 mg bid, and loxapineHCl 25 mg qhs. A year later, in 2005, her endocrinologist prescribed cabergoline to treat the pituitary adenoma to replace the bromocriptine she had been receiving for five years.

Ms. L's next hospitalization five years later was the first of four in one year. She was sad and tearful, felt her psychiatric medications were not working, and had stopped them. Her last dose of flupenthixoldecanoate had been several months prior. She was experiencing impaired concentration, memory, and energy. She had cut her hair bizarrely, felt she had psychic abilities, and reported that her vision was impaired. Ms. L was highly regressed, initially refusing treatment, but once restarted on her oral medications, she recompensated quickly. She remained with behaviours such as rocking and circular hand movements and returned to the group home never having remembered or understood why she had suddenly left it. She was discharged on divalproex sodium 500 mg thrice daily (tid), procyclidineHCl 5 mg bid, and loxapineHCl 25 mg qhs for her psychosis and continued cabergoline 0.5 mg twice weekly for the pituitary adenoma. 

Three weeks after discharge, Ms. L was picked up by the police in an apartment lobby where she had spent the night. Ms. L was confused and catatonic, again suffering impaired insight, judgement, and memory. She spoke of being possessed, hearing voices, and having visual hallucinations. She was discharged with ziprasidone 40 mg bid added to her prescription and a followup with her endocrinologist. An MRI showed that her microadenoma had shrunk. Four weeks later, without apparent provocation, Ms. L quietly walked out of her group home during dinner. She was found by police roaming barefoot. She again had serious memory problems and disorganized thinking. Her ziprasidone was increased to 60 mg bid, the loxapine decreased to 12.5 mg, and electroencephalography (EEG) was suggested to rule out seizures in an effort to explain her memory loss.

### 2.2. Psychotherapeutic Intervention

 I (K. Drury) had become Ms. L's therapist during her seventh admission. The hospitalization lasted 3 weeks during which we worked almost exclusively on sustaining eye contact. My clinical impression of this young woman was that she had been deprived of healthy attachments and as a result had retreated deep into herself. In our work together, she slowly began to respond to my demands for her to sit up straight, look at me in the eye, and stop circling her fingers. At discharge, Ms. L agreed to come to the hospital twice a week to meet with me. During these sessions, Ms. L often spoke of her impaired vision and related how one of the indicators of her improvement was that she “starts to see again.” In fact, this metaphor alerted us to the possibility of an interaction between her pituitary tumor and psychosis. We had had no evidence of the possibility of compression of the optic chiasm by the pituitary tumor. Ms. L seemed to respond well to a consistent, structured relationship. She spoke about the trauma of her parents' divorce, which she understood to be the precipitating event to her illness. She was not able to stay with this topic, and it was clear that it was very distressing to her. Two weeks into our outpatient work, Ms. L was readmitted after she suddenly got up from the dinner table and walked barefoot out of her group home. As in the previous admissions, she was disoriented, disorganized, and unable to explain what had happened or why she was in hospital. Despite her persistent cloudy mental state, she spent many hours in occupational therapy painting, knitting, and making jewellery, which she took great pride in wearing. She wrote some poetry. She was able to cease the circular finger movements, make consistent eye contact, and articulate some of her feelings.

### 2.3. Psychiatric Treatment

Three months later, Ms. L was admitted feeling there was a devil in the group home, and that the chicken that was served in the home was human meat, and the vegetables were bewitched. Ms. L's language had been disorganized for several days. Her sleep was impaired, and she was shaking more and circling her fingers. As with the last admission, there was no known environmental precipitant. Ms. L was not eating as she was paranoid about the hospital food. Her EEG was normal.

M. Sigman had had concerns about the possible psychotic side effects of the dopamine agonist cabergoline used to treat the pituitary tumor and hyperprolactinemia. The general physician to the psychiatric ward had been encouraged to consult the involved endocrinologist on this issue, and this resulted in the cabergoline being discontinued. Ms. L was reorganized enough to begin to eat and participate in milieu treatment. She had trials of both ziprasidone and aripiprazole but remained psychotic in her thinking, for example, getting orders from the devil. Clozapine was started, and the psychosis began to remit. The endocrinologist felt Ms. L could be kept off the cabergoline if it impaired her mental status. While M. Sigman was on vacation, a psychiatry resident took over Ms. L's care until she was discharged with a diagnosis of psychosis secondary to substance (cabergoline) versus exacerbation of psychosis secondary to substance. Ms. L continued to improve and has not been hospitalized since. Was it getting off the cabergoline or starting the Clozaril that ultimately led to her improved state, or, as we believe, a combination of both?

## 3. Discussion

Ms. L presented with a constellation of risk factors for mental illness; her mother suffers from schizophrenia, Ms. L was the victim of sexual, physical, and emotional abuse, and she was without social support. The severity and complexity of Ms. L's symptoms made it very difficult to unearth deleterious interactions between her medications. An important issue that evolved over the course of her admissions was the need for more discussion among medical specialities within the hospital network. This was not easily achieved. For example, after several admissions, there had been a written request that the outpatient psychiatrist consults with the endocrinologist about the psychosis, inducing effects of cabergoline, but the patient was admitted again before this ever occurred. With increasing specialization, medical treatment has moved in the direction of parsing the individual into treatable components, rather than in the direction of teamwork and interacting systems. This could have contributed to the prolongation of Ms. L's difficulties. The World Federation for Mental Health highlighted the following factors that often result in people with a mental illness not receiving adequate health care in relation to their overall health needs: social marginalization and isolation, discrimination, and the separation of health care for physical illness from mental health care [4]. There is increasing evidence for the interaction between physical and mental health [5]. Additionally, it has been found that standardized mortality rates increased threefold in patients with schizophrenia particularly with reference to diseases of the cardiovascular, respiratory, digestive, endocrine, and nervous systems [6]. Separation of physical and mental health care often fragments the total care offered to individuals suffering from a mental illness perhaps explaining why physical illness often goes undetected in psychiatric patients [7]. 

There is not an extensive literature on patients with both psychosis and a pituitary tumor. Acute psychosis has been reported elsewhere in a woman with a known prolactinoma [8] and in three patients with psychoses and concomitant prolactin-secreting pituitary tumors [9]. A review of the literature on bromocriptine and psychosis concluded that confusion, hallucinations, and delusions have often been reported with the use of bromocriptine, [10, 11]. In addition, cabergoline-induced psychotic exacerbation in schizophrenic patients has been discussed elsewhere [12]. 

 Ms. L's prolactin level on her first hospital admission was 125.1 (normal is 20–25 ng/mL). Such an elevation can occur from stress, medication, a pituitary tumor, and so forth [13]. Ms. L had experienced all of these. It is possible that at her first admission both the pituitary tumor and high prolactin level and her life stressors led to Ms. L's psychotic illness. As cabergoline replaced bromocriptine as the treatment of choice for the prolactinoma, Ms. L's tumor shrank and her prolactin level came to within normal limits. This change is reliable indication that though Ms. L was not always taking her antipsychotic medication, she was most likely taking the medication that targeted her pituitary tumor. After Ms. L stopped her intramuscular antipsychotic injections feeling they were not working, she had four admissions to hospital in rapid succession. She had become acutely disorganized and was very impaired in her eating behaviours, her judgement, and memory. 

 It is possible that the dopamine agonists bromocriptine and cabergoline which Ms. L had received for ten years for the pituitary adenoma promoted her psychotic thinking for which she was treated with multiple sequential psychiatric medications (dopamine antagonists). Other authors have reported prolactinoma growth with risperidone treatment and suggest using other antipsychotic medications, which do not affect prolactin secretion [1]. It has been reported that psychotic patients treated with aripiprazole showed a lower liability toward hyperprolactinemia [14]. Aripiprazole did not help Ms. L's psychosis. Others have recommended the use of clozapine, which is what Ms. L was prescribed on her last admission [9]. A successful treatment of schizophrenia with no elevation of serum prolactin levels using a combination of olanzapine and quetiapine was reported in a patient who could not tolerate clozapine [15].

## 4. Conclusion

This case study highlights both the importance of understanding the role of prolactin in psychotic illness and of an interdisciplinary team approach when patients present with multiple and complex symptoms.
